# Anti-IgE Qb-VLP Conjugate Vaccine Self-Adjuvants through Activation of TLR7

**DOI:** 10.3390/vaccines4010003

**Published:** 2016-01-21

**Authors:** Bassel Akache, Risini D. Weeratna, Aparna Deora, Jennifer M. Thorn, Brian Champion, James R. Merson, Heather L. Davis, Michael J. McCluskie

**Affiliations:** 1 Pfizer Vaccine Immunotherapeutics, Ottawa Laboratories, 340 Terry Fox Drive, suite 200, Ottawa, ON K2K 3A2, Canada; bassel.akache@pfizer.com (B.A.); risini.weeratna@pfizer.com (R.D.W.); heather.davis@pfizer.com (H.L.D.); 2 Pfizer Biotherapeutics Pharmaceutical Sciences, St. Louis, MO 63017, USA; aparna.deora@pfizer.com (A.D.); jennifer.thorn@pfizer.com (J.M.T.); 3 Pfizer Vaccine Immunotherapeutics, La Jolla, CA 92121, USA; brian.champion@psioxus.com (B.C.); james.merson@pfizer.com (J.R.M.)

**Keywords:** Qb, VLP, TLR7, bacteriophage, IgE, asthma, CpG

## Abstract

Qb bacteriophage virus-like particles (Qb-VLP) are utilized as carriers to enhance immune responses to weakly or non-immunogenic antigens such as peptides and haptens. Qb-VLPs are formed through the self-assembly of multiple Qb capsid protein monomers, a process which traps a large amount of bacterial RNA in the core of the VLP. Bacterial RNA is known to activate the innate immune system via TLR 7 and 8 found within the endosomes of certain immune cells and has been shown to contribute to the immunogenicity of Qb-VLP vaccines. Herein, we evaluated an anti-IgE vaccine comprised of two IgE peptides (Y and P) conjugated to Qb-VLP (Qb-Y and Qb-P, respectively) for *in vitro* stimulation of human PBMCs and *in vivo* immunogenicity in mice. The *in vitro* secretion of IFN-α from human PBMCs exposed to Qb-Y is consistent with TLR7 activation. Immunization of mice with the IgE peptide Qb-VLP conjugates induced high titers of anti-IgE antibodies in wild-type mice, but significantly lower titers in TLR7 knockout mice, supporting the self-adjuvanting role of the RNA. Inclusion of alum and alum/CpG as adjuvants partially or completely compensated for the lack of TLR7 activation in TLR7-deficient mice. Our study demonstrates the key role that TLR7 plays in the immunogenicity of the IgE peptide Qb-VLP conjugate vaccine.

## 1. Introduction

Qb bacteriophage virus-like particles (Qb-VLPs) have been used in therapeutic vaccines as a conjugate carrier for antigens that are on their own non-immunogenic (nicotine hapten) or only weakly immunogenic (e.g., peptides derived from angiotensin II, interleukin-1β) and these have been tested in clinical studies [1,2,3].

The Qb VLP comprises 180 subunits of the Qb capsid protein such that when the VLP is used for conjugation of antigens (e.g., haptens or peptides), they are presented to the immune system in a highly ordered repetitive array, which can be effective for the induction of immune responses and breaking B cell tolerance [4]. The Qb-VLP self-assembly relies on the formation of an initiation complex composed of an RNA strand and a few molecules of capsid protein; this complex then allows for cooperative interaction between the remaining capsid proteins [5]. As such, a significant amount of RNA (~25% of VLP mass) becomes encapsulated [6]. Bacterial RNA is known to act as a pathogen-associated molecular pattern recognized by TLR7 and TLR8 found on certain innate immune cells (e.g., B cells, myeloid-derived dendritic cells) and as such could serve as an auto-adjuvant for Qb VLP-conjugate antigens [7]. Indeed, Qb-VLPs devoid of RNA through treatment with RNase can generate lower titers of anti-Qb IgG antibodies (Ab) in mice, with a shift from IgG2c to IgG1 compared to VLP with RNA [8]. In addition, the efficacy of an RNA-containing AP205 bacteriophage VLP-influenza M2e vaccine in mice depended on its ability to generate M2e-specific IgG2c Abs mediated through TLR7 activation. Specifically, although total IgG titers were not impacted in TLR7 knockout mice immunized with the M2e-VLP vaccine, there were significantly lower IgG2c titers and increased susceptibility to infection after viral challenge than in wild-type controls [9].

We have developed an anti-IgE vaccine for the treatment of allergic asthma and rhinitis comprising two separate but admixed human IgE peptide Qb-VLP conjugates (Qb-Y and Qb-P) as antigen and aluminum hydroxide as adjuvant. In preclinical models, the vaccine induced high titers of anti-human IgE Abs and also IgE lowering in non-human primates, in which sequence homology to humans is high [10]. The peptides, labeled as P and Y, are derived from different loops of the C3 domain of IgE that binds to the high affinity FcεRI receptor on mast cells, basophils and eosinophils. The peptide Y includes the epitope recognized by omalizumab, the humanized anti-IgE monoclonal Ab approved for treatment of uncontrolled moderate to severe allergic asthma and antihistamine-resistant chronic idiopathic urticaria [11]. Herein, we investigated the role of the RNA (TLR7 agonist) contained within the Qb-VLP carrier in the immunogenicity of our anti-IgE vaccine.

## 2. Material and Methods

### 2.1. Vaccine Antigens

IgE peptide Qb-VLP conjugates (Qb-Y and Qb-P) were prepared by a 2-step process, consisting of the activation of the Qb-VLP (Pfizer, St. Louis, MO, USA) with succinimidyl 6-beta-maleimidopropionamido hexanoate (SMPH; SAFC, Gillingham, UK) followed by conjugation with peptide. SMPH in DMSO (Sigma-Aldrich, St. Louis, MO, USA) was added to 100 mg of Qb-VLP (3 mg/mL in 20 mM sodium phosphate (J.T. Baker, Center Valley, PA, USA), 150 mM NaCl (J.T. Baker) (pH 7.2)) at either 10× molar excess (for peptide P: ADSNPRGVSAYLSRPSPGGC) or 4.25× molar excess (for peptide Y: QCRVTHPHLPRALMRS). The level of SMPH was chosen to target a peptide load of 2.5 and 1.5 peptides per Qb-P and Qb-Y monomer, respectively. The solution was incubated at 15 °C for 5 h with continuous mixing. The activated Qb-VLP was then purified and buffer exchanged into 100 mM sodium phosphate, 300 mM NaCl (pH 6.8) by ultrafiltration/diafiltration (UF/DF) using a Sartorius Slice UF system installed with a Biomax 300 kD membrane. The purified activated Qb-VLP was diluted to 1 mg/mL with 100 mM sodium phosphate, 300 mM NaCl (pH 6.8) prior to the addition of peptide at a 7× molar excess. Conjugation proceeded at 15 °C for 1.5 h with continuous mixing. The conjugated Qb-VLP was then purified and buffer exchanged into 100 mM sodium phosphate, 200 mM NaCl (pH 7.2) by UF/DF using a Sartorius Slice UF system installed with a Biomax 300 kD membrane. Sucrose (J.T. Baker) at 140 mg/mL and PS20 (J.T. Baker) at 0.2 mg/mL were added to the purified IgE peptide Qb-VLP conjugates. The concentration was adjusted to 2.5 mg/mL and the conjugates were frozen at −80 °C until further use. Peptide load (number of peptides attached per Qb monomer) was determined by SDS-PAGE.

### 2.2. Adjuvants

Aluminum hydroxide (alum) was obtained in the form of Alhydrogel “85” (Brenntag Biosector, Frederikssund, Denmark). The B Class CpG ODN (CpG) of sequence 5' TCG TCG TTT TTC GGT GCT TTT 3' was synthesized with a nuclease-resistant phosphorothioate backbone (Avecia, Milford, MA, USA) as described previously [12].

### 2.3. In Vitro Stimulation of Human Immune Cells and IFN-α ELISA

Human PBMCs were freshly isolated from whole blood and cultured as previously described [13]. Cells were initially incubated with or without chloroquine (Invivogen, San Diego, CA, USA) for 30 min, prior to the addition of Qb-Y or loxoribine (7-allyl-7,8-dihydro-8-oxoguanosine; Sigma-Aldrich) as a positive control. Chloroquine and loxoribine were dissolved at a concentration of 100 mM in water and DMSO, respectively. The concentration of human IFN-α in the cellular supernatants collected 18–20 h post-stimulation was determined by sandwich ELISA following the manufacturer’s instructions (PBL Biomedical, Piscataway, NJ, USA).

### 2.4. Animals

Wild-type (WT) and TLR7 knockout (KO) C57BL/6 mice were obtained from Taconic (Hudson, NY, USA). The TLR7 KO model has been described previously [14]. All procedures performed on animals in this study were in accordance with regulations and guidelines reviewed and approved by the Pfizer Institutional Animal Care and Use Committee and were conducted in facilities fully accredited by AAALAC International.

### 2.5. Immunization of Mice

Mice (*n* = 16) were immunized by intramuscular (i.m.) injection into the left tibialis anterior muscle on days 0, 28 and 56 with the IgE peptide Qb-VLP conjugates (admixed, 10 μg each of Qb-Y and Qb-P), either without adjuvant or in combination with alum (50 μg Al^3+^) or alum (50 μg Al^3+^)/CpG (50 μg) made up to a total volume of 50 μL with PBS (Sigma-Aldrich). Dose levels were selected based on data from previous studies to induce strong Ab responses in wild-type mice. Animals were bled on day 70 and recovered serum was used for quantification of IgE and Qb-specific immune responses.

### 2.6. Anti-IgE/Qb Ab ELISA

The levels of anti-IgE or anti-Qb Abs in mouse serum were quantified by ELISA using 384–well MaxiSorp ELISA plates (Thermo Fisher Scientific, Waltham, MA, USA) coated overnight at 4 °C with 25 µL of 5 µg/mL human IgE (Abbiotec, San Diego, CA, USA) or 1 µg/mL Qb-VLP in PBS (Life Technologies, Grand Island, NY, USA). Plates were washed three times with PBS, and then blocked with 80 µL 1% bovine serum albumin (Sigma-Aldrich) in PBS for 1 h at room temperature (RT). After the plates were washed three times with PBS, 3.162-fold serially diluted samples in PBS with 1% bovine serum albumin were added in 25 µL volumes and incubated for 1 h at RT. After five washes with PBS/0.05% Tween 20 (Sigma-Aldrich), 25 µL of goat anti-mouse IgG-HRP (1:30,000, Abcam, Cambridge, MA, USA), goat anti-mouse IgG1-HRP (1:4000, Southern Biotech, Birmingham, AL USA) or goat anti-mouse IgG2c-HRP (1:4000, Southern Biotech) was added for 1 h at RT. After five washes with PBS/0.05% Tween 20, 25 µL/well of the substrate tetramethylbenzidine (TMB, Mandel Scientific, Guelph, ON, Canada) was added. Plates were developed for 20 min at RT in the dark. The reaction was stopped with 1N H_2_SO_4_, 12.5 µL/well. Bound IgG Abs were detected spectrophotometrically at 450 nm. Titers for IgG in serum were defined as the dilution that resulted in an absorbance value (OD 450) of 1 and grouped data was presented as geometric mean titer (GMT).

### 2.7. Statistical Analysis

Data were analyzed using GraphPad Prism (GraphPad Software, Inc., San Diego, CA, USA). Statistical significance of the difference between groups was calculated by 1-factor ANOVA followed by post-hoc analysis. Differences were considered to be not significant with *p* > 0.05.

## 3. Results and Discussion

### 3.1. Qb-VLPs Induce IFN-α Secretion in Human PBMCs

**Figure 1:**
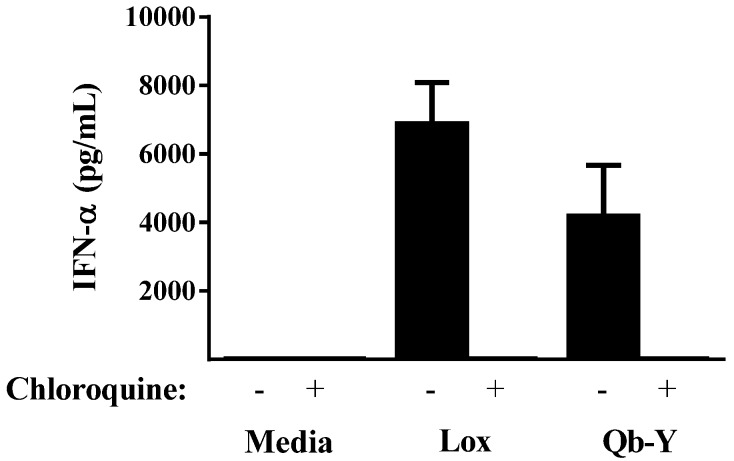
Stimulation of IFN-α secretion in human PBMCs. Cultured cells were stimulated with either 300 µM loxoribine or 75 µg/mL of Qb-Y in the absence or presence of 10 µM chloroquine for 16–20 h. The levels of IFN-α in the supernatants were measured and compared to those obtained with unstimulated cells (*n* = 4). The assay’s lower limit of quantification (12.5 pg/mL) was used when the amount of IFN-α was too low to be detected. The results are representative of those obtained from a similar experiment using PBMCs from a second human donor.

IFN-α secretion is a well-recognized marker of TLR7 activation, with immune cells (e.g., PBMCs) secreting high levels upon exposure to ssRNA or small molecule TLR7 agonists such as loxoribine [7,15]. Incubation of human PBMCs with Qb-Y resulted in the secretion of IFN-α (Figure 1). As expected, this immune activation was abolished by chloroquine, a known antagonist of endosomal TLRs including TLR7 [16].

### 3.2. Immune Responses to the IgE Peptide Qb-VLP Conjugates in WT Mice

**Figure 2:**
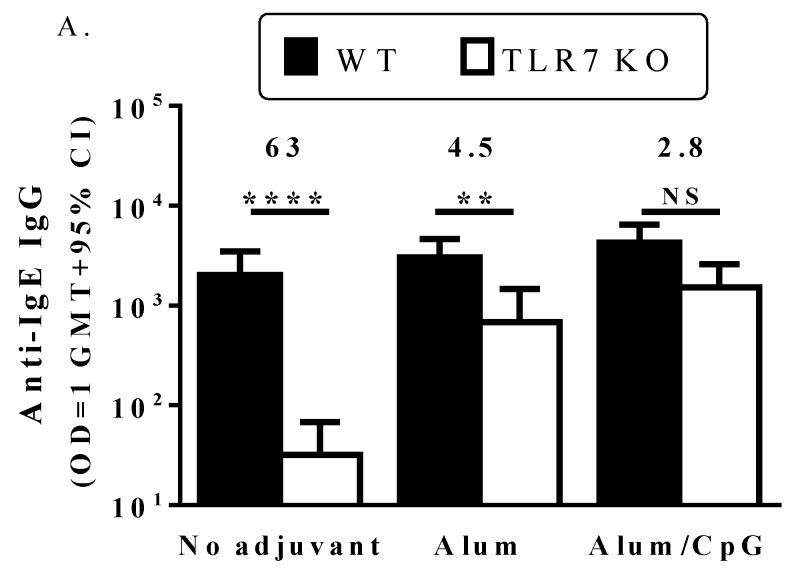

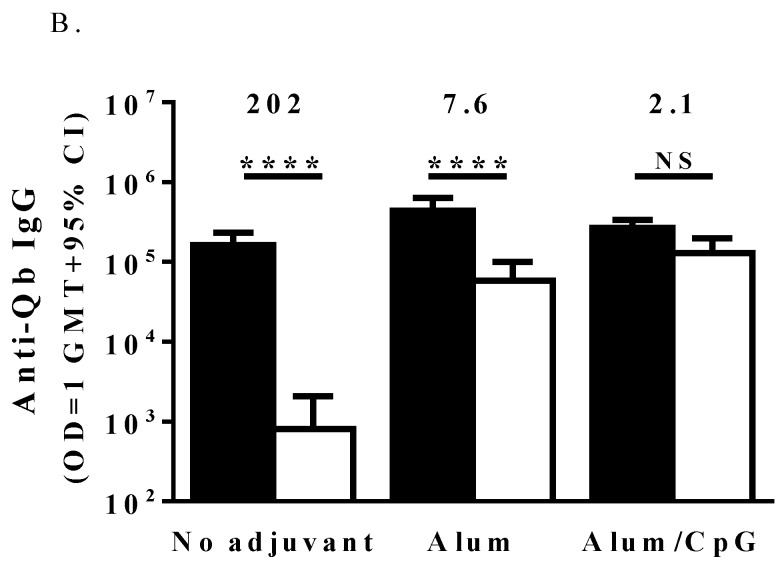
Anti-IgE and anti-Qb titers in WT and TLR7 KO mice. C57BL/6 mice (*n* = 16/group) were immunized with 20 µg IgE peptide Qb-VLP conjugates (10 μg each of Qb-Y and Qb-P) without adjuvant or with alum (50 µg) or alum/CpG (50 µg each) on days 0, 28 and 56. Animals were bled on day 70 and plasma analyzed for anti-IgE (**A**) or anti-Qb (**B**) IgG Abs by ELISA. The fold-decrease in IgG titers between wild-type and TLR7 KO mice is indicated above the bars. The depicted data is from one animal study, but similar results were obtained in a separate study. Two and four stars indicate a significance of *p* < 0.01 and *p* < 0.0001, respectively. NS: Not significant.

In WT mice, the IgE peptide Qb-VLP conjugates induced high titers of anti-IgE IgG Abs, with similar results whether or not adjuvants (alum or alum/CpG) were included (Figure 2A). The lack of alum adjuvant effect points to the high inherent immunogenicity of the VLP. Furthermore, the lack of additive or synergistic effects for TLR7 (Qb VLP) and TLR9 (CpG) agonists was not unexpected since both TLRs work through the same MyD88-dependent signaling pathway [14,17] and their similar modes of action may preclude any enhancement of immune responses when combined. Lack of synergy between TLR7 and 9 agonists has been reported previously [18]. In addition, co-stimulation by TLR7/8 and TLR9 agonists have been shown to down-regulate immune responses *in vitro* [19].

### 3.3. TLR7 Activity Mediates the Induction of Anti-IgE Immune Responses by the IgE Peptide Qb-VLP Conjugates

Results in the TLR7 KO mice were quite different. First, the non-adjuvanted conjugate vaccine induced titers of anti-IgE Ab ~60-fold lower than those in WT mice, demonstrating the dependence on TLR7 activity for inherent immunogenicity of the VLP (Figure 2A; *p* < 0.0001). Similar differences were observed in the anti-Qb IgG titers (Figure 2B; *p* < 0.0001). While this supports the findings of Bessa *et al.* [8] that Qb VLP RNA plays a role in inducing anti-Qb IgG Abs, it is in contrast to observations from other studies with RNA-containing bacteriophage VLP vaccines. Jegerlehner *et al.* concluded that anti-Qb IgG levels were similar between animals immunized with VLPs devoid of or containing RNA [20]. Likewise, a vaccine consisting of an RNA-containing bacteriophage, AP205, conjugated to an influenza epitope M2E, generated similar levels of anti-M2e IgG titers in WT and TLR7 KO mice [9]. In the latter studies, lack of TLR7 activation during vaccination led to a decrease in antigen-specific IgG2a/c titers, but there was also a concomitant increase in IgG1 Abs. The differing observations between studies may be due to differences in the antigens administered, antigen dose, dose schedule and/or endpoints used. In order to further explore the role that RNA has in Qb-VLP mediated immune activation, we attempted to generate IgE peptide Qb-VLP conjugates devoid of RNA. However, degradation of the RNA resulted in significant changes to the overall structure of the VLPs such that we were unable to synthesize stable RNA-free VLPs. This likely reflects the key role that RNA has not only in self-adjuvanting Qb-VLPs but also in providing structural integrity.

The second important difference from studies in WT mice was that there was a benefit to adding extraneous adjuvants. Although alum on its own significantly enhanced Ab titers (*p* < 0.0001), they were still significantly lower (~4.5-fold) than in WT mice (*p* < 0.01). However, with the addition of both alum and CpG, anti-IgE titers were restored to WT levels, indicating that the CpG could replace the role of the RNA (Figure 2). The third difference was that with the non-adjuvanted vaccine, TLR7 KO mice had significantly lower anti-IgE IgG2c titers (*p* < 0.0001) and IgG2c:IgG1 ratios (*p* < 0.05) than WT mice, indicative of a more Th2-biased response (Table 1). TLR7 activation has previously been shown to induce Th1-biased immune responses characterized by the generation of Abs predominantly of the IgG2a/c subtype in mice [8,9,20]. While the alum-adjuvanted vaccine formulation induced strong anti-IgE IgG1 titers in the TLR7 KO mice, the IgG2c titers remained low. This was not surprising as alum is typically associated with Th2 responses (IgG1 > IgG2) in mice. While the exact mechanism of alum as adjuvant has not been fully elucidated, it is believed to be a combination of a repository or depot effect, a pro-phagocytic effect, and activation of the pro-inflammatory NLRP3 pathway [21]. As was seen for total IgG, the use of alum/CpG as adjuvant increased IgG2c titers in TLR7 KO mice as compared to unadjuvanted mice or mice with alum alone as adjuvant, which was not surprising as CpG is a strong Th1-biased adjuvant with similar effects to RNA [22]. The IgG2c GMT titer observed with the alum/CpG adjuvanted formulation in the KO mice was similar to that obtained with the unadjuvanted formulation in the WT mice (2183 *vs.* 2528) and slightly lower than obtained with the alum/CpG adjuvanted formulation in WT mice (2183 *vs.* 7440), although this difference was not statistically significant (*p* > 0.05). However, while the titers are similar, the activation of TLR7 and/or TLR9 by the different vaccine formulations may have generated Abs of differing avidity or functionality. The key role that Ab avidity plays in determining Ab function has previously been demonstrated in clinical studies evaluating an angiotensin-Qb VLP conjugate vaccine (AngQb) to treat hypertension [3,23]. While initial testing of a three dose regimen (0, 4, 12 week) reduced blood pressure in subjects, subsequent attempts to drive higher Ab titers with increased number and frequency of doses (0, 2, 4, 6, 8, 10 week) resulted in loss of efficacy due to lower Ab avidity [1,24]. Although there may be differences in the avidity or functionality of Abs generated in this study via activation of TLR7 and/or TLR9, confirmation of such differences requires further investigation.

**Table 1:** IgG1 *vs.* IgG2c ratios of anti-IgE Abs in WT and TLR7 KO mice. IgG1 and IgG2c titers were determined by ELISA and correspond to the serum dilution that resulted in an absorbance value (OD 450) of 1. GMT: Geometric mean titer; CI: Confidence interval.

Mouse Strain	Vaccine Formulation	IgG1		IgG2c		IgG2c/IgG1	
		GMT	95% CI	GMT	95% CI	GMT	95% CI
WT	Unadjuvanted	130	58.9–287.2	2528	1567.6–4075.4	19.43	7.75–48.73
	Alum	1010	571.7–1785.5	3408	2124.1–5469.4	3.37	1.56–7.28
	Alum/CpG	797	351–1809.6	7440	4599.5–12,034.2	9.33	4.57–19.09
TLR7 KO	Unadjuvanted	144	44.6–465.1	11	9.2–12.6	0.07	0.02–0.23
	Alum	3185	1666.5–6087.9	92	28.1–304.1	0.03	0.01–0.08
	Alum/CpG	2859	1689.8–4835.5	2183	939.4–4635.2	0.76	0.43–1.37

## 4. Conclusions

We have demonstrated herein the importance of TLR7 in the generation of strong antibody responses to our IgE peptide Qb-VLP conjugate vaccine. It is likely that the RNA contained within Qb VLPs not only serves as a scaffold allowing the assembly of the particles, but may also act as an innate immune activator that serves as an auto-adjuvant influencing the magnitude and quality of the immune response through TLR7 activation.
